# Extraction of Phytochemicals from Maypole Apple by Subcritical Water

**DOI:** 10.3390/foods11213453

**Published:** 2022-10-31

**Authors:** Menghsuan Tsai, Li Zhu, Shinya Maeda, Tao Wang, Wahyu Diono, Motonobu Goto, Hideki Kanda

**Affiliations:** 1 Department of Materials Process Engineering, Nagoya University, Furocho, Chikusa, Nagoya 464-8603, Japan; 2 New Industry Creation Hatchery Center, Tohoku University, 6-6-10 Aoba, Aramaki, Aoba-ku, Sendai 980-8579, Japan; 3 Super Critical Technology Centre Co., Ltd., Kuwana 511-0838, Japan

**Keywords:** Maypole apple, subcritical water, procyanidin B2, 5-caffeoylquinic acid, epicatechin

## Abstract

The Maypole apple is a new, promising species of small apples with a prominent flavor and deep red flesh and peel. This study divided Maypole apples into outer flesh, inner flesh, and peel, and used subcritical water at 100–175 °C for 10–30 min to extract various phytochemicals (procyanidin B2 (PB2), 5-caffeoylquinic acid (5CQA), and epicatechin). The obtained Maypole apple extracts and extraction residues in this work were analyzed using a SEM, HPLC, FT-IR, and UV-Vis spectrophotometer. Under different subcritical water extraction conditions, this work found the highest extraction rate: to be PB2 from the peel (4.167 mg/mL), 5CQA (2.296 mg/mL) and epicatechin (1.044 mg/mL) from the inner flesh. In addition, this work regressed the quadratic equations of the specific yield through ANOVA and found that temperature is a more significant affecting factor than extraction time. This aspect of the study suggests that phytochemicals could be obtained from the Maypole apple using the new extraction method of subcritical water.

## 1. Introduction

The Maypole apple is a type of crabapple that has been evaluated as a potential pollinizer for major apple cultivars [1]. It has been reported that phytochemical composition and contents significantly vary between the apple peel and flesh [2,3]. Because of the dark red color of the flesh and peel of Maypole apples, they are expected to contain a considerable amount of phytochemicals. Phytochemicals have been reported to have different benefits for human health, such as controlling immune and inflammatory responses, inhibiting cancer cell growth, and preventing lipid oxidation [3,4,5]. This work focuses on the extraction of phytochemicals such as procyanidin B2 (PB2), 5-caffeoylquinic acid (5CQA), and epicatechin from Maypole apples.

Procyanidins are polymers or oligomers of flavonoids, which are commonly found in various fruits and plants [6]. PB2 is an antioxidant that is primarily found in procyanidin extracts from grape seeds [7,8]. Some apple varieties also accumulate PB2 in their flesh and peel [9]. PB2 has been reported to have various beneficial effects, including antitumor [10], anti-DNA damage [11], anti-inflammatory [12], and hair growth-activating effects [13].

5CQA is a caffeic acid ester that is also known as chlorogenic acid. 5CQA is commonly found in plants, including fruits, vegetables, and coffee [14,15]. In addition, a high amount of 5CQA has been found in apples [16]. 5CQA is a dietary polyphenolic compound with various significant therapeutic effects, including antioxidant, antibacterial, hepatoprotective, cardioprotective, anti-inflammatory, antipyretic, neuroprotective, anti-obesity, antiviral, antimicrobial, antihypertensive, and central nervous system stimulation effects [16,17,18].

Epicatechin is a natural flavonoid found in green tea [19]. It has been reported to have a substantial antioxidant effect that contributes to its therapeutic effect. Epicatechin has been reported to exhibit beneficial effects in controlling sugar levels in diabetic patients [20], show antioxidant effects [21], prevent cardiovascular disease [22], improve exercise performance [23], and inhibit cancer [24].

Traditionally, organic solvents such as methanol and ethanol have been used to extract phytochemical compounds from various natural products [25,26,27,28]. Methanol is toxic to human health, and some regions and cultures prohibit the use of ethanol in food processing, necessitating the use of alternative solvents. Moreover, the latest research shows that even small amounts of alcohol can affect health [29]. Water, in contrast, is a green solvent that is non-toxic to human health and the environment, and has no cultural problems. In addition, replacing organic solvents with water in phytochemical compound extraction is expected to lead to significant cost reduction. In the present study, water was used to extract PB2, 5CQA, and epicatechin from Maypole apples.

If water is heated above 374 °C and pressurized above 22 MPa, it becomes supercritical. Supercritical water has low viscosity, low surface tension, and a high diffusion coefficient [30]. In addition, supercritical water could behave as a nonpolar solvent, meaning that ionic compounds are poorly soluble in it [31]. Thus, supercritical water can serve as a good solvent for nonpolar compounds [32,33,34].

Subcritical water is milder than supercritical water. This study defines subcritical water as water under high pressure at 100–374 °C. Although some bioactive compounds could be degraded at a high temperature [35], it has been reported that tannic acid, as a typical polyphenolic compound, has major degradation at temperatures above 230 °C [36]. Therefore, the use of subcritical water up to 175 °C is mild for this work. It is reported that subcritical water contains a range of hydrogen and hydroxide ions [37]. In addition, previous studies have indicated that the dielectric constant of subcritical water at 250 °C in the liquid state (ε = 27) is similar to that of methanol (ε = 33) and ethanol (ε = 24) at 25 °C [38,39]. This indicates that subcritical water may have good solubility for natural products, comparable to that of methanol or ethanol. In addition, with the increase in water temperature to 200 °C, the surface tension of water decreases by nearly 50%, making it easier for the subcritical water to wet the raw materials and thus increasing the extraction efficiency [40,41]. Furthermore, the pressurized hot water is expected to effectively break the cell wall, especially for the peel of the Maypole apple, and extract the inner biological components. Owing to its various advantages, subcritical water has been reported to be a new and promising extraction medium for the extraction of various phytochemicals from various natural products [42,43,44].

The present study aimed to investigate the optimal temperature and time for the subcritical water extraction of PB2, 5CQA, and epicatechin from various parts of Maypole apples. This study also provides more experimental data for future use of subcritical water extraction to extract different phytochemicals from natural resources.

## 2. Materials and Methods

### 2.1. Materials

Maypole apples, used as the raw material, were picked from the Nakahira farm in Matsukawa-Cho, Nagano Prefecture, Japan, in early September. The pure water used in this study was distilled using a distillation apparatus (Auto Still WS 200; Yamato Scientific Co. Ltd., Tokyo, Japan). The PB2 standard was purchased from Fuji Film Wako Junyaku Co. Ltd., Osaka, Japan. (-)-Epicatechin was purchased from Hayashi Kasei Co., Ltd. Nagoya, Japan. 5CQA (98% content) was purchased from Kanto Kagaku Co., Ltd., Tokyo, Japan. Acetic acid (99.5% purity) and acetonitrile (99.8% purity) were purchased from Fujifilm Wako Pure Chemical Industries Ltd., Osaka, Japan.

**Figure 1:**
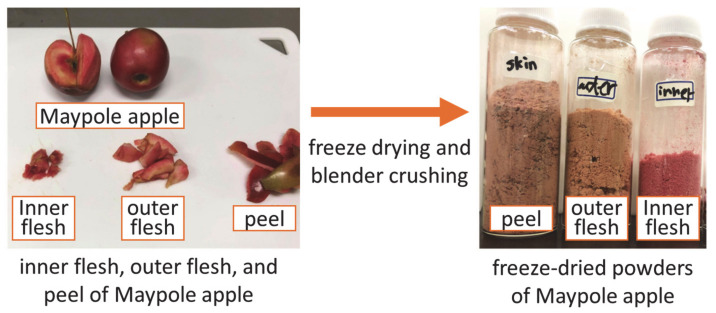
Pre-processing of different parts of Maypole apples.

Figure 1 shows a flow chart illustrating the preprocessing of Maypole apples. Apples were washed with tap water and peeled using a peeler. Subsequently, the apples were divided into three parts: the peel, outer flesh, and inner flesh, which were freeze-dried (Eyela FDU-1200; Rikakikai Co., Ltd., Tokyo, Japan) for 24 h and crushed into powder. After freeze-drying, the moisture contents of the peel, outer flesh, and inner flesh were 6.53 wt.%, 9.43 wt.%, and 10.94 wt.%, respectively, which was tested using a halogen moisture meter (65g0001g; AS ONE Corp., Osaka, Japan).

### 2.2. Methods

**Figure 2:**
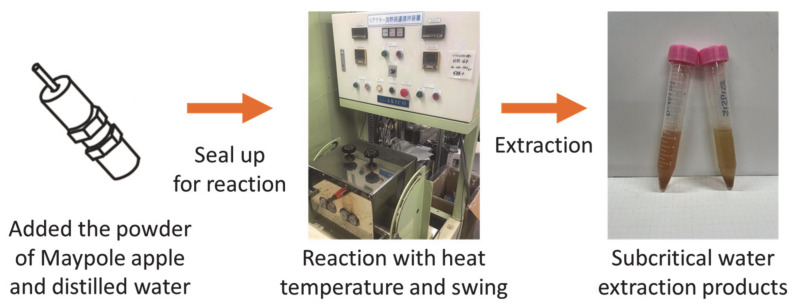
Extraction of different parts of the Maypole apple by subcritical water.

Figure 2 shows the batch high-temperature swing device (AKICO Co., Ltd., Tokyo, Japan). A sample of 0.2 g freeze-dried Maypole apple powder and 7 mL distilled water were added to an 8 mL SUS-316 reactor and hermetically sealed. The reactor was then loaded into an electric furnace (NMF-13AD, Isuzu Corporation, Tokyo, Japan) and heated to the desired temperature (100–175 °C). The swing device was set to 60 times/min. After 10–30 min of reaction, the reactor was removed from the electric furnace and quenched in a cold water bath with water flow under ambient conditions. Cooling took 10–20 min, depending on the reaction temperature. After the reactor was cooled, it was removed from the water bath, and its lid was removed so as to collect the extract and residue. In order to prevent bioactive compounds from reacting with light, the extracts were wrapped in aluminum foil and stored at <6 °C for further characterization.

### 2.3. Analytical Methods

#### 2.3.1. Scanning Electron Microscopy (SEM) Analysis and Imaging of Residues

The morphologies of the residues were observed using SEM (S-4300, Hitachi, Tokyo, Japan). The samples were sputter-coated with gold (RMC-Eiko RE vacuum coater, Eiko Engineering Co., Ltd., Tokyo, Japan) at 100 millitorrs under 7 mA for 300 s prior to SEM in order to capture a zoomed-in image of the samples.

#### 2.3.2. High-Performance Liquid Chromatography (HPLC)

Subcritical extracts were centrifuged at 2500 rpm for 10 min prior to HPLC. The centrifuged extracts were added with 5 μL of 10 g/L *o*-cresol (99% purity, Fujifilm Wako Pure Chemical Industries Co., Ltd., Osaka, Japan) as an intermediate marker to 1 mL of supernatant. Ten microliters of the obtained solution was sampled using an HPLC autosampler (SIL-10 AF, Shimadzu Corporation, Kyoto, Japan) and injected into the HPLC apparatus. The flow rate was 1.0 mL/min, and the wavelength was set to 280 nm for PB2 [45,46], 5CQA [47,48], and epicatechin [49,50].

Because it is difficult to resolve all individual peaks, a gradient method with two mobile phases [51] was used. Mobile phase A consisted of 2.5 vol.% aqueous acetic acid, and mobile phase B was acetonitrile. The gradient conditions were set as follows: 0 min A–B (97:3), 5 min A–B (91:9), 15 min A–B (84:16), 33 min A–B (64:36), 38 min A–B (0:100), 48 min A–B (97:3), and 60 min A–B (97:3).

HPLC pumps A (LC10-AD, Shimadzu Corporation, Kyoto, Japan) and B (LC20-AD, Shimadzu Corporation, Kyoto, Japan) were used. The separation column STR ODS-II (4.6 mm × 250 mm; Shinwa Chemical Industry, Kyoto, Japan) was used at a temperature of 40 °C. The HPLC detector was an SDP-M10A diode array detector (Shimadzu Corp., Kyoto, Japan).

#### 2.3.3. Fourier Transform Infrared (FT-IR) Spectroscopy

FT-IR spectroscopy (Spectrum Two, PerkinElmer Ltd., Buckinghamshire, UK), in the wavenumber range of 400–4000 cm^−1^, was used to analyze Maypole apple solids and subcritical water extraction residues.

#### 2.3.4. Total Phenolic Content (TPC) Analysis

The TPC was determined using the Folin-Ciocalteu reagent. Specifically, 1 mL of the extract was added to 1 mL of the Folin-Ciocalteu reagent in a dark room. After 3–5 min, 7 mL of distilled water and 1 mL of 7.5 wt.% sodium carbonate were added to the mixture. The solution was placed in a dark room for 3 h until all reactions were completed. The absorbance of the solution was measured at 765 nm on a single-beam UV-Vis spectrophotometer (V-550, JASCO, Tokyo, Japan) [52,53]. This procedure was performed three times for each sample, and the average absorbance was calculated.

## 3. Results and Discussions

### 3.1. Appearance of the Extracts

**Figure 3:**
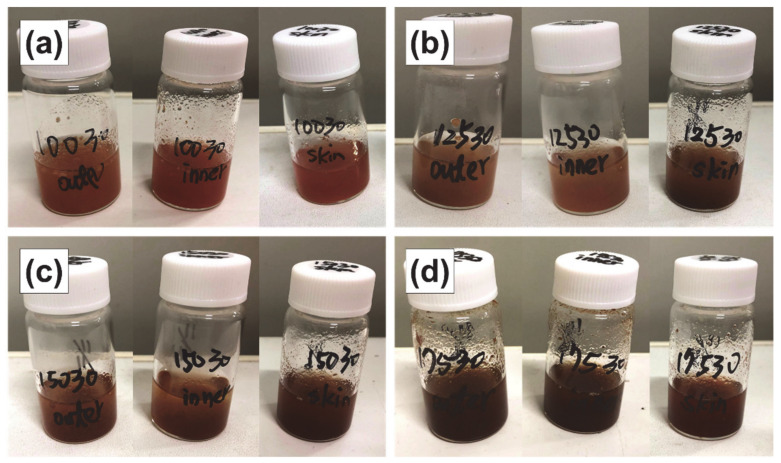
Subcritical water extracts of the outer flesh, inner flesh, and peel of Maypole apples at (**a**) 100 °C, (**b**) 125 °C, (**c**) 150 °C, and (**d**) 175 °C for 30 min.

As discussed below, an extraction time of 30 min is optimal; therefore, the extract obtained under these conditions is shown. As shown in Figure 3, with the increase in extraction temperature from 100 to 175 °C, the color of the subcritical water extracts became darker; thus, the total extracted content is expected to increase with extraction temperature.

### 3.2. SEM and Imaging of the Residues

**Figure 4:**
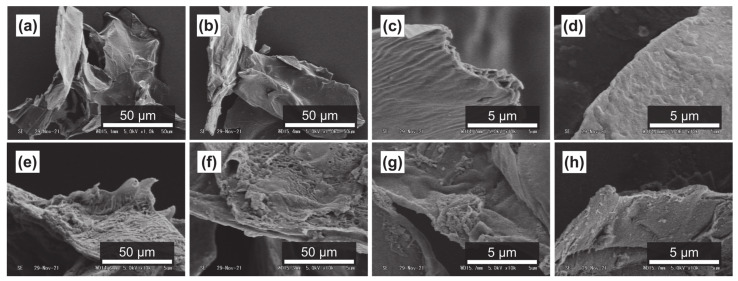
SEM images of the outer flesh of Maypole apples: (**a**,**b**) raw under ×1.0k magnification; (**c**,**d**) raw under ×10.0k magnification; (**e**) treated with 100 °C subcritical water for 10 min; (**f**) treated with 150 °C subcritical water for 10 min; (**g**) treated with 175 °C subcritical water for 10 min; and (**h**) treated with 175 °C subcritical water for 20 min.

**Figure 5:**
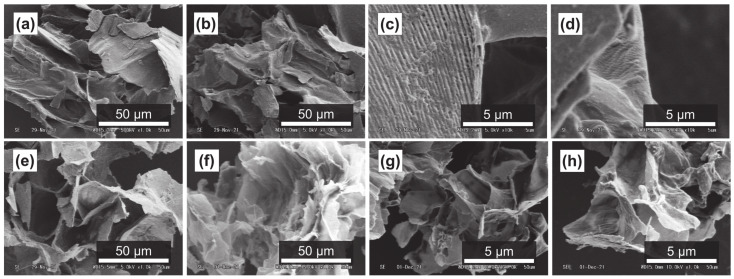
SEM images of the inner flesh of Maypole apples: (**a**,**b**) raw under ×1.0k magnification; (**c**,**d**) raw under ×10.0k magnification; (**e**) treated with 100 °C subcritical water for 10 min; (**f**) treated with 100 °C subcritical water for 30 min; (**g**) treated with 175 °C subcritical water for 10 min; and (**h**) treated with 175 °C subcritical water for 30 min.

**Figure 6:**
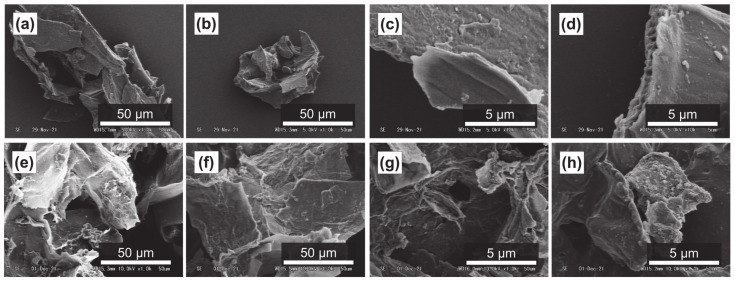
SME images of the peel of Maypole apples: (**a**,**b**) raw under ×1.0k magnification; (**c**,**d**) raw under ×10.0k magnification; (**e**) treated with 100 °C subcritical water for 10 min; (**f**) treated with 100 °C subcritical water for 30 min; (**g**) treated with 175 °C subcritical water for 10 min; and (**h**) treated with 175 °C subcritical water for 30 min.

It has been reported that subcritical water can effectively decompose polysaccharides in natural products [54,55]. Figure 4a–d, Figure 5a–d and Figure 6a–d show SEM images of the outer flesh, inner flesh, and peel of Maypole apples, respectively, before subcritical water treatment. These images can be used to observe the morphology of the cell wall, which is primarily composed of multiple polysaccharides [56]. As shown in Figure 4e–h, Figure 5e–h and Figure 6e–h, the SEM images indicate that under high temperatures and pressures, subcritical water can effectively break the structure of cells and penetrate them. Subcritical water is expected to directly dissolve active ingredients inside the cells of Maypole apples.

### 3.3. HPLC Results

HPLC showed the characteristic peaks of PB2, 5CQA, and epicatechin. We obtained PB2, 5CQA, and epicatechin yields by calculating the peak areas under the calibration curves of standard samples. After calculating the weight of the powder used, the specific yield was calculated using Equation (1).

(1) $$\mathrm{specific}\;\mathrm{yield}\;\left[\mathrm{mg}/g\right]=\frac{\mathrm{yield}\;\left[\mathrm{mg}\right]}{\mathrm{weight}\;\mathrm{of}\;\mathrm{dried}\;\mathrm{Maypole}\;\mathrm{apple}\;\mathrm{powder}\;\left[g\right]}$$

#### 3.3.1. Yield of PB2

**Figure 7:**
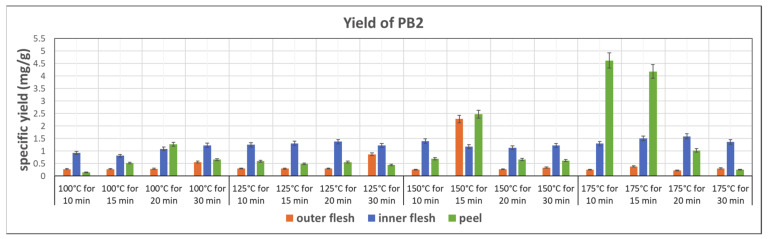
Specific yield of the subcritical extraction of PB2 from the outer flesh, inner flesh, and peel of Maypole apples at different temperatures and extraction times.

Figure 7 shows the specific yield of PB2 in subcritical water extracts from the outer flesh, inner flesh, and peel, under different conditions. The peel of Maypole apples yielded the maximum amount of PB2. In addition, the yield of PB2 is strongly related to the extraction temperature. Generally, higher temperatures result in higher PB2 contents. The highest PB2 content (4.167 mg/g) can be observed in the extract obtained at 175 °C and 10 min from the peel. Because of the thick epidermal cell walls, subcritical water at the highest tested temperature (175 °C) and high pressure broke down tissue fibers and extracted PB2 the most effectively. However, the results also show that bioactive PB2 easily decomposes at 175 °C. Therefore, subcritical water extraction of PB2 should be accomplished within 10–15 min.

#### 3.3.2. Yield of 5CQA

**Figure 8:**
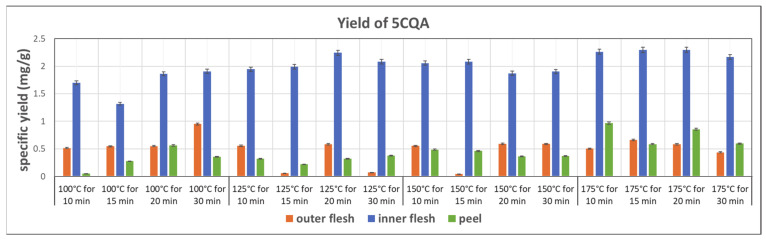
Specific yield of the subcritical extraction of 5CQA from the outer flesh, inner flesh, and peel of Maypole apples at different temperatures and extraction times.

The yields of 5CQA in the subcritical water extraction from the outer flesh, inner flesh, and peel are shown in Figure 8. In general, compared to other parts of Maypole apples, the inner flesh yields more than three times more 5CQA in the extraction with subcritical water. In addition, 5CQA did not significantly decompose at 175 °C within 30 min. The highest 5CQA content (2.296 mg/g) can be observed in the extract from the inner flesh obtained at 175 °C and 20 min.

#### 3.3.3. Yield of Epicatechin

**Figure 9:**
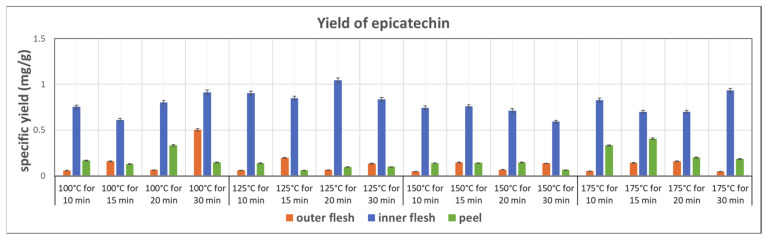
Specific yield of the subcritical extraction of epicatechin from the outer flesh, inner flesh, and peel of Maypole apples at different temperatures and extraction times.

Figure 9 shows the yields of epicatechin obtained in the subcritical water extraction of the outer flesh, inner flesh, and peel, under different conditions. The inner flesh of Maypole apples yielded remarkably high epicatechin contents, with the highest extraction yield of 1.044 mg/g obtained at 125 °C and 20 min. The yield of epicatechin in the inner flesh was double that in the outer flesh and peel. Therefore, different parts of the Maypole apple should be used for extracting different phytochemical compounds.

#### 3.3.4. Analysis of Variance (ANOVA)

This work utilizes ANOVA to evaluate the correlation between the extraction rate of phytochemicals in Maypole apples and subcritical water extraction conditions. Temperature (100–175 °C) and extraction time (10–30 min) were selected as two independent factors of subcritical water extraction. From the data shown in Figure 7, Figure 8 and Figure 9, the highest extract rate of the three types of phytochemicals from Maypole apples were observed, which were PB2 from peel extracts and 5CQA and epicatechin from the inner flesh extracts, respectively.

Table 1 presents the ANOVA of these three sets of data. The F-test result indicates that the subcritical water extraction affecting factors of PB2, 5CQA, and epicatechin are temperature (°C) > Time (min). In addition, the lower *p*-value indicates that temperature has a more significant predictability in extraction rate than extraction time.

Additionally, this work found the predictive quadratic models for predicting yields of PB2 in peel ((Equation (2), *p*-value = 0.0074, <0.05) and 5CQA in inner flesh ((Equation (3), *p*-value = 0.0456, <0.05) were statistically significant. In these models, *f*(*x*, *y*) is the specific yield of the extracted liquid (mg/mL), *x* is the subcritical water temperature (°C), and *y* is the reaction time (min).

(2) $$f\left(x,\;y\right)=1.12532-0.08275\;x+0.44846\;y-0.0033xy+0.00061x^{2}-0.00149y^{2}$$

(3) $$f\left(x,\;y\right)=-0.58506+0.02323\;x+0.0593\;y-0.00035xy-0.00004x^{2}-0.0002y^{2}$$

In addition, this work also demonstrates other significant quadratic regression equations, including the specific yield of 5CQA from the peel ((Equation (4), *p*-value = 0.0038, <0.05), and the specific yield of epicatechin from the peel ((Equation (5), *p*-value = 0.0456, <0.05). In these equations, *f*(*x*, *y*) is the specific yield of the extracted liquid (mg/mL), *x* is the subcritical water temperature (°C), and *y* is the reaction time (min).

(4) $$f\left(x,\;y\right)=0.96307-0.02382\;x+0.06762\;y-0.00038xy+0.00013x^{2}-0.00039y^{2}$$

(5) $$f\left(x,\;y\right)=1.47682-0.02431\;x+0.02674\;y-0.00014xy+0.0001x^{2}-0.00029y^{2}$$

Therefore, it is considered that different phytochemicals can be obtained from different parts of Maypole apples. In addition, the temperature (°C) of the subcritical water is expected to be a more important factor than the extraction time (min).

### 3.4. Analysis of FT-IR Spectra

**Figure 10:**
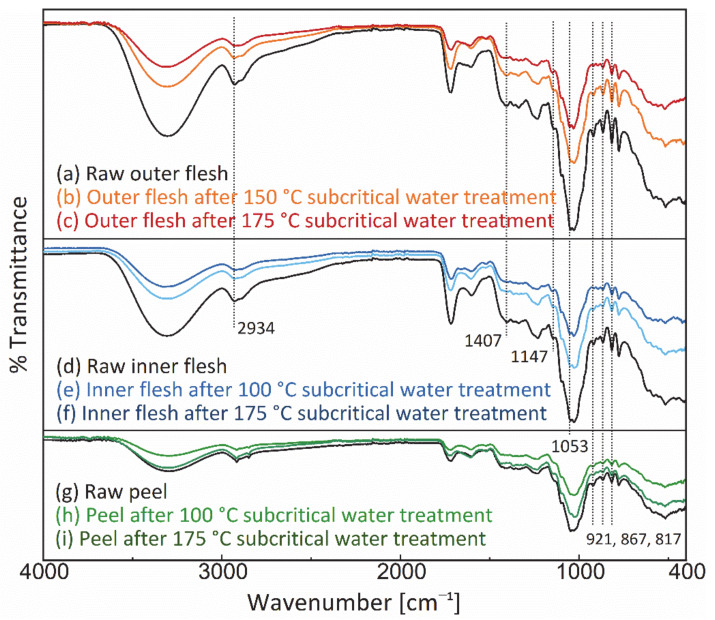
FT-IR spectrum of the outer flesh, inner flesh, and peel of Maypole apples.

Figure 10 shows the FT-IR spectra of the Maypole apple outer flesh, inner flesh, and peel before and after subcritical water treatment at 175 °C. In the FT-IR spectra of the outer and inner flesh, the moderate intensity band at 2934 cm^−1^, the strong band at 1147 cm^−1^, and the highest intensity band at 1053 cm^−1^ are characteristic bands of glucose; they correspond to C–H asymmetric stretching, C–O stretching, and C–O, C–C–C asymmetric tensile vibration [57]. In addition, the moderately intense band at 1407 cm^−1^ was attributed to the deformation vibrations of O-C–H, C–O–H, and C–C–H in fructose [58]. In addition, the moderately intense bands at 921, 867, and 817 cm^−1^ were assigned to the C–H deformation vibrations of fructose and the C–H stretching and CH_3_ rocking vibrations of pectin (a polysaccharide), respectively [59].

In the present study, no new characteristic peaks were observed in the FT-IR spectra of different parts of Maypole apples, thus suggesting that subcritical water extraction at 175 °C did not generate new significantly observable or potentially toxic chemicals. Additionally, the peak intensities of the extraction residue were lower than those of the respective raw materials, indicating that subcritical water can extract the characteristic phytochemical components of Maypole apples.

### 3.5. TPC Analysis

**Figure 11:**
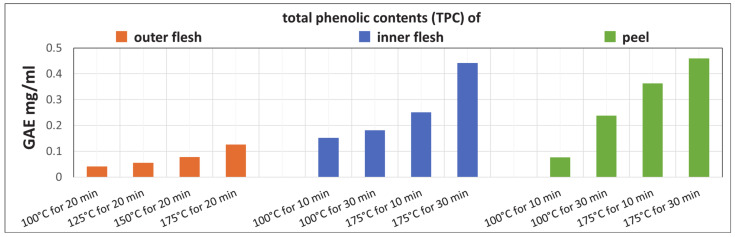
TPC after subcritical extraction of the outer flesh, inner flesh, and peel of Maypole apples at different temperatures and extraction times.

Figure 11 shows that the TPCs of the Maypole apple outer flesh, inner flesh, and peel extracts increase with the extraction temperature and time. In addition, the TPCs of the raw inner flesh and peel (which have a dark red color) are significantly higher than that of the outer flesh (which is lighter in color).

The results indicate that extraction from Maypole apples with subcritical water at 175 °C for 30 min yields high TPC. In addition, in subcritical water environments up to 175 °C, high-molecular-weight polyphenols are expected to decompose, forming other compounds (phenolic, etc.) that become extractable.

## 4. Conclusions

In this study, subcritical water was successfully used to extract phytochemicals, including PB2, 5CQA, and epicatechin, from various parts of Maypole apples. SEM images showed that under high temperatures and pressures, subcritical water could effectively break the structure of cells and penetrate into the Maypole apple cells. The results of HPLC showed that the phytochemical components of various parts of the apple could be extracted from subcritical water under different conditions. Particularly, the content of PB2 was the highest (4.167 mg/g) in the extract from the peel obtained at 175 °C and 10 min extraction time. The 5CQA content was the highest (2.296 mg/g) in the extract from the inner flesh obtained at 175 °C and 20 min. The epicatechin content was the highest (1.044 mg/g) in the extract from the inner flesh obtained at 125 °C and 20 min. The concentration of phenolic compounds in the extract was the highest at 175 °C and 20–30 min. Moreover, the ANOVA results showed that the temperature of the subcritical water extraction is expected to be a more critical factor than the extraction time.

According to the results of this study, subcritical water can effectively extract phytochemicals from Maypole apples. Subcritical water extraction does not require the use of organic solvents, and is environmentally friendly. This work further provides more experimental data of the extraction of phytochemicals using subcritical water.

## Figures and Tables

**Table 1:** ANOVA for experimental parameters.

	Factors	F-Test	*p*-Value
PB2 from peel	Temperature (°C)	7.6615	0.0199
	Time (min)	4.8738	0.0518
5CQA from inner flesh	Temperature (°C)	12.0804	0.006
	Time (min)	0.2321	0.6403
Epicatechin from inner flesh	Temperature (°C)	0.2888	0.6027
	Time (min)	0.1325	0.7234

## Data Availability

Data is contained within the article.
